# Nutrient stoichiometric and resorption characteristics of the petals of four common urban greening Rosaceae tree species

**DOI:** 10.3389/fpls.2023.1201759

**Published:** 2023-06-15

**Authors:** Dan Song, Shuting Liu, Lide Fan, Jinyan Yang, Haifang Li, Yujie Xia, Yuwu Li

**Affiliations:** ^1^ College of Landscape Architecture and Forestry, Qingdao Agricultural University, Qingdao, China; ^2^ Academy of Dongying Efficient Agricultural Technology and Industry on Saline and Alkaline Land in Collaboration with Qingdao Agricultural University, Dongying, China

**Keywords:** Rosaceae, fresh petals, petal litter, nutrient resorption, stoichiometry, Qingdao

## Abstract

**Objective:**

Nutrient resorption efficiency and stoichiometric ratios are important strategies for understanding plants. The present study examined whether or not the nutrient resorption process of plant petals is similar to that of leaves and other vegetative organs, as well as the nutrient restriction status of the whole flowering process of plants in urban ecosystems.

**Methods:**

Four Rosaceae tree species, *Prunus yedoensis* Matsum, *Prunus serrulata* var. *lannesiana, Malus micromalus* Makino, and *Prunus cerasifera* ‘Atropurpurea’, were selected as urban greening species to analyze the contents of C, N, P, and K elements in the petals and their stoichiometric ratios and nutrient resorption efficiencies.

**Results:**

The results show interspecific differences in nutrient contents, stoichiometric ratios, and nutrient resorption efficiency of the fresh petals and petal litter of the four Rosaceae species. The nutrient resorption process was similar to that of the leaves before the petals fell. The nutrient contents of petals were higher than that of leaves at the global level, but the stoichiometric ratio and nutrient resorption efficiency of petals were lower. According to the "relative resorption hypothesis", N was limiting during the entire flowering period. The nutrient resorption efficiency of petals was positively correlated with nutrient variation. The correlation between the nutrient resorption efficiency of petals with nutrient content and stoichiometric ratio of petal litter was stronger.

**Conclusion:**

The experimental results provide scientific basis and theoretical support for the selection, scientific maintenance and fertilization management of Rosaceae tree species in urban greening.

## 1 Introduction

In the long-term evolution process, plants have formed a series of mechanisms and strategies to adapt to the changing environment. One of the important, nutrient resorption is important for nutrient recycling and reuse under the pressures of resource crisis caused by environmental changes and the need for a stable balance of nutrients to maintain the normal physiological activities of plants (Sterner and Elser, 2002; Fan et al., 2015; Xie et al., 2020; Li et al., 2021), thus increasing the chances of plant survival and species continuation (Zhao et al., 2006). Nutrient resorption is the transfer and transport of nutrients to other tissues of plants before senescence and litterfall (Killingbeck, 1986; Aerts, 1996; Lu et al., 2018); it provides energy and nutrients for new tissues and prolongs the retention time of nutrients in plants, thereby reducing the process of plant dependence on external nutrients (Covelo et al., 2008; Zhao et al., 2010; Vergutz et al., 2012). Nutrient resorption is a protective mechanism by which plants improve nutrient use efficiency in soil nutrient-poor environments (Yuan et al., 2006; Jiang et al., 2017). It also reduces the nutrient loss caused by litter decomposition and leaching (May and Killingbeck, 1992). Therefore, nutrient resorption plays an important role in plant adaptation to stress, nutrient preservation, enhancement of competitiveness and productivity, and maintenance of the stoichiometric balance of chemical elements in plants (Brant and Chen, 2015; Lu et al., 2018).

Carbon (C), nitrogen (N), phosphorus (P), and potassium (K), as essential life elements for the structure of plant somatic cells and growth and development, have a strong coupling effect (Ågren, 2004) and play an important role in various physiological functions of plant growth and adaptation to the environment. Reiners (1986) first proposed the theory of ecological stoichiometry, and many researchers have since studied and expanded the application of ecological stoichiometry theory in consumer-driven nutrient cycling (Sterner, 1990; Urabe et al., 1995), biological nutrient limitation (Koerselman and Meuleman, 1996; Aerts and Chapin, 1999), forest succession and decline (Wardle et al., 2004), and ecosystem nutrient supply and demand balance (Schade et al., 2005; Zeng and Chen, 2005). Among such applications, plant C, N, P, and K stoichiometry is an important means of studying the energy cycle and element balance of terrestrial ecosystems. C:N, C:P, and C:K can directly reflect the nutrient utilization characteristics of plants to a certain extent. N:P, N:K, and K:P can be used as indicators of the supply of soil nutrients to plant growth (Li et al., 2010; Yang et al., 2021). Recent years have seen many studies based on plant leaves. However, studies on nutrient content and element stoichiometric characteristics of flower organs, such as petals, during flowering are still rare. In particular, few reports have been done on whether there are similar nutrient resorption processes in plant petals and other organs accompanying this process. Studying the nutrient resorption and stoichiometric characteristics of plant petals is helpful for understanding the nutrient cycling process of urban ornamental flowering tree species.

There are approximately 124 genera and more than 3300 species of Rosaceae, which are widely distributed in the world but more common in the north temperate zone. China is the distribution center of Rosaceae, with about 50 genera and more than 1000 species (Su et al., 2015; Zou et al., 2019). Rosaceae, playing an important role in urban landscaping all over the world, are mostly ornamental flower plants, whose fruits, flowers, branches, and leaves have high ornamental value and strong ecological adaptability. Nutrient management of urban plants is the key to the management of urban green space and is also the basis for the selection, management, and ecological evaluation of urban landscaping plants (He, 2002).

Therefore, we chose four common Rosaceae species in northern China, *Prunus yedoensis* Matsum, *Prunus serrulata* var. *lannesiana*, *Malus micromalus* Makino, and *Prunus cerasifera* ‘Atropurpurea’, to clarify the C, N, P, and K nutrient contents, stoichiometric characteristics and their relationship with nutrient resorption of the fresh petals and petal litter of them, to study the petal nutrient resorption strategies during the flowering, and to judge whether there is an obvious nutrient resorption process in petals. The major scientific questions are as follows: (1) How did the nutrient content of the four Rosaceae species change during the short period of flowering and falling? (2) Is there a nutrient resorption process in petals similar to that in leaves? Do petals have greater nutrient resorption capacity than leaves? (3) What is the relationship between the nutrient content and stoichiometric ratio of the fresh petals and petal litter and nutrient resorption efficiency?

## 2 Materials and methods

### 2.1 Study site

The study was conducted in a green space at the Qingdao Agricultural University, Shandong Province, China (36°19’38 “N, 120°24’19” E), located in the north temperate zone and with a temperate oceanic monsoon climate (warm and humid). The site has a flat terrain and mainly brown loam soil. According to China Weather Network in, the annual average temperature of the area was 13.5°C in 2022, with August being the hottest month (25.5°C) and January the coldest (0°C). The annual average precipitation was 662.1 mm, with the most rainfall from June to August. The terrain of the test site is flat, and the soil is mainly brown loam.

### 2.2 Sample collection

Four Rosaceae species were commonly planted in the study site, including *Prunus yedoensis* Matsum, *Prunus serrulata* var. *lannesiana*, *Malus micromalus* Makino, and *Prunus cerasifera* ‘Atropurpurea’ were selected for the study. Three well-developed plants of each tree species were selected as the research objects. The basic information of the trees is shown in **Table 1**.

**Table 1:** Basic information of four Rosaceae tree species.

Species	Tree Age (year)	Tree Height (m)	Diameter at Breast Height (cm)	Tree Crown (m)
*Prunus yedoensis* Matsum	22	5.55 ± 0.43	17.40 ± 1.18	6.10 ± 0.65
*Prunus serrulata* var. *lannesiana*	22	5.75 ± 0.19	23.03 ± 2.43	6.72 ± 0.19
*Malus micromalus* Makino	20	5.73 ± 0.26	23.10 ± 1.40	7.19 ± 0.57
*Prunus cerasifera* ‘Atropurpurea’	20	5.61 ± 0.20	24.67 ± 1.45	4.94 ± 0.32

Using herring-shaped escalators and high-branch shears, petals of the same weight were collected from branches in four directions, east, west, south and north of the crown of each sample tree, and mixed into a 100g samples of petals at the full bloom period (April 2022). These samples were immediately placed in Ziplock bags and brought to the laboratory, with three replicates collected per species.

Petal litter samples were collected during the whole flowering period (April to May 2022) of the selected tree species. Two 5 m × 2.5 m collection nets were set on both sides of the trunk of each tree species. Petals were collected from the nets at regular intervals until they fell completely. Each time, petal litters from the two collection nets of the same tree were mixed and placed in a Ziplock bag as a replicate (three replicates for each species).

### 2.3 Chemical analysis

After removing dust and impurities, the plant samples, including petals and petal litter, were dried to a constant weight in an oven at 60°C and ground to powder; this powder was passed through a 100-mesh sieve (0.149 mm) and stored until further analysis. The C and N contents in the plant samples were determined using an elemental analyzer (Primacs SNC 100-IC, Skalar Analytical B.V., Netherlands). The P and K were determined using an ICP-AES (iCAP6300, Thermo Fisher Scientific, USA) following HNO_3_-HClO_4_ digestion and leaching. Further, the nutrient resorption efficiency (NuRE) was calculated as follows (Lu et al., 2018):


NuRE=(1−NulitterNufresh)×100%

where Nu _litter_ indicates the nutrient content of petal litter (g·kg^-1^), and Nu _fresh_ means the nutrient content of fresh petals (g·kg^-1^).

Relative resorption efficiency (RR) was calculated as follows (Han et al., 2013):


RR=NRE−PRE

where NRE means the nitrogen resorption efficiency (%), and PRE means the phosphorus resorption efficiency (%). When RR≈0, plants are limited by N, P balance, or both. RR>0, plant growth was limited by N. RR<0, plant growth was limited by P.

Finally, the C, N, P, and K stoichiometric ratios of the fresh petals and petal litters were expressed as C:N, C:P, C:K, N:P, N:K, and K:P, and the element content ratio was adopted.

### 2.4 Statistical analyses

All data were represented as mean ± standard error. The independent sample *t-*test was used to compare the differences in N, P, and K contents, stoichiometric ratios, and nutrient resorption efficiencies between the fresh petals and petal litter of the same tree species. One-way analysis of variance (ANOVA) and Duncan’s multiple range test (α = 0.05) were used to compare between-species differences in each parameter. Origin 2021 was used to conduct linear fitting analysis on the relationships between nutrient resorption efficiencies of N, P, and K and the corresponding nutrient contents of the fresh petals and petal litter. Pearson correlation analysis (two-tailed) was used to analyze the correlation between nutrient resorption efficiency and the stoichiometric ratios of the fresh petals and petal litter, with Origin 2021 used to make correlation heat maps.

## 3 Results

### 3.1 C, N, P, and K contents of fresh petals and petal litter

**Figure 1:**
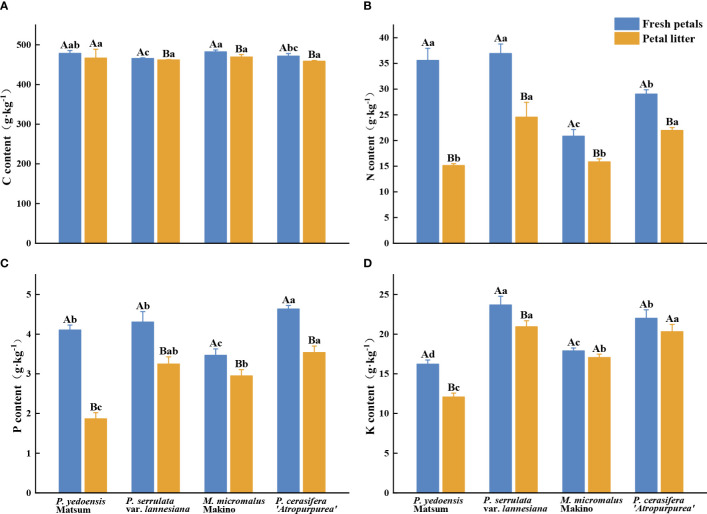
C, N, P, and K content of petals in fresh petals and petal litter of four Rosaceae species. Different uppercase letters indicate significant differences between fresh petals and petal litter of the same tree species (*P<* 0.05), and different lowercase letters indicate significant differences between different tree species of fresh petals or petal litter (*P*< 0.05).

The average C, N, P, and K contents of fresh petals of the four Rosaceae tree species were 465.67–482.33 g·kg^-1^, 20.84–36.93 g·kg^-1^, 3.47–4.63 g·kg^-1^, and 16.22–23.68g·kg^-1^, respectively (**Figure 1**). The content of C in fresh petals of *P. yedoensis* Matsum was significantly higher than that of *P. serrulata* var. *lannesiana* (*P*< 0.05), and that in *M. micromalus* Makino was significantly higher than that of *P. serrulata* var. *lannesiana* and *P. cerasifera ‘Atropurpurea’* (*P*< 0.05) (**Figure 1A**). The contents of N in fresh petals of *P. yedoensis* Matsum and *P. serrulata* var. *lannesiana* were significantly higher than those of *M. micromalus* Makino and *P. cerasifera ‘Atropurpurea’* (*P*< 0.05) (**Figure 1B**). Meanwhile, the P content in the fresh petals of *M. micromalus* Makino was significantly lower than those of the other three species (*P*< 0.05) (**Figure 1C**). The content of K in fresh petals of *P. serrulata* var. *lannesiana* was the highest, while that of *P. yedoensis* Matsum was the lowest (*P*< 0.05) (**Figure 1D**).

The C, N, P, and K content in fallen petal litter were 458.67–469.33 g·kg^-1^, 15.13–24.56 g·kg^-1^, 1.87–3.54 g·kg^-1^, and 12.08–20.93g·kg^-1^, respectively (**Figure 1**). No significant differences were found in C content in petal litter among the four Rosaceae species (*P* > 0.05) (**Figure 1A**). However, the N contents of the petal litter of *P. serrulata* var. *lannesiana* and *P. cerasifera ‘Atropurpurea’* were significantly higher than that of *P. yedoensis* Matsum and *M. micromalus* Makino (*P*< 0.05) (**Figure 1B**). The P content of the petal litter of *P. yedoensis* Matsum was considerably lower than that of the other three species (*P*< 0.05) (**Figure 1C**). The K content of the petal litter of *P. serrulata* var. *lannesiana* and *P. cerasifera ‘Atropurpurea’* was the highest, while that of *P. yedoensis* Matsum was the lowest (*P*< 0.05) (**Figure 1D**).

The analysis also showed that the nutrient contents were higher in the fresh petals than in the petal litter for the four Rosaceae species. Except for *P. yedoensis* Matsum, the other three species had significantly higher C contents in the fresh petals than that in petal litter (*P*< 0.05) (**Figure 1A**). Similarly, the contents of N and P in the petal litter of the four Rosaceae species were significantly lower than those in the fresh petals (*P*< 0.05) (**Figures 1B, C**). The contents of K in the fresh petals were markedly higher than those in the petal litters of *P. yedoensis* Matsum and *P. serrulata* var. *lannesiana* (*P*< 0.05) (**Figure 1D**).

### 3.2 C, N, P, and K stoichiometric ratios in fresh petals and petal litter

**Figure 2:**
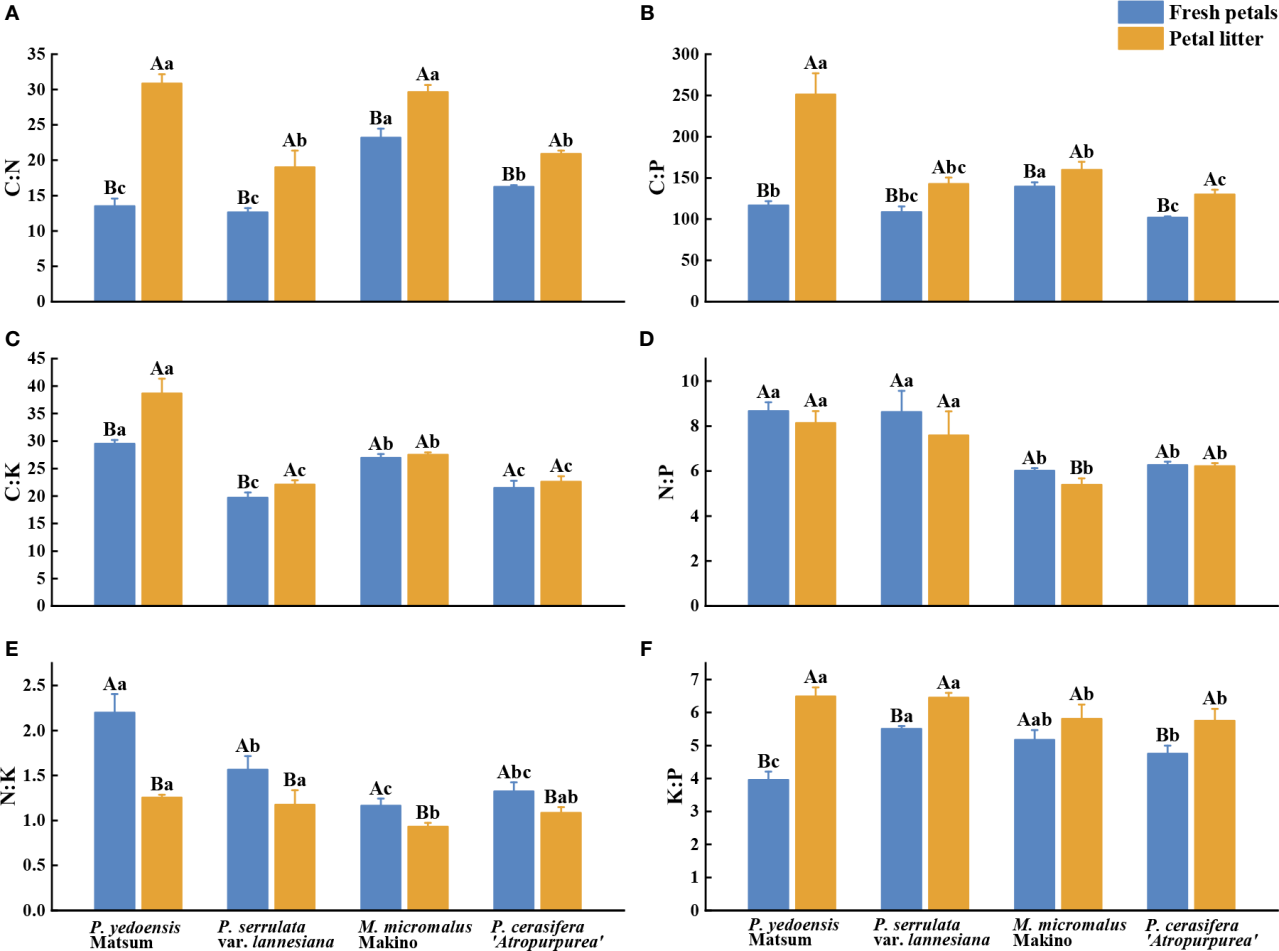
C, N, P, and K stoichiometric ratios of petals in fresh petals and petal litter of four Rosaceae species. Different uppercase letters indicate significant differences between fresh petals and petal litter of the same tree species (*P<* 0.05), and different lowercase letters indicate significant differences between different tree species of fresh petals or petal litter (*P*< 0.05).

The average C:N, C:P, C:K, N:P, N:K, and K:P ratios in the fresh petals of the four Rosaceae species were 12.63–23.19, 101.74–139.30, 19.70–29.51, 6.01–8.67, 1.17–2.20, and 3.96–5.5, respectively (**Figure 2**). The C:N and C:P of fresh petals of *M. micromalus* Makino were significantly the highest among the four species (**Figures 2A, B**), and the C:K and N:K of *P. yedoensis* Matsum were statistically significantly the highest (*P*< 0.05) (**Figures 2C, E**). The N:P of *P. yedoensis* Matsum and *P. serrulata* var. *lannesiana* was markedly higher than that of *M. micromalus* Makino and *P. cerasifera ‘Atropurpurea’* (**Figure 2D**), while the K:P of *P. yedoensis* Matsum was significantly lower than that of the other three species (*P*< 0.05) (**Figure 2F**).

Meanwhile, the C:N, C:P, C:K, N:P, N:P, N:K, and K:P of the petal litters of the four Rosaceae species were 19.00–30.84, 129.86–251.22, 22.09–38.68, 5.39–8.14, 0.93–1.25, and 5.75–6.49, respectively (**Figure 2**). The C:N of the petal litters of *P. yedoensis* Matsum and *M. micromalus* Makino were significantly higher than those of *P. serrulata* var. *Lannesiana* and *P. cerasifera ‘Atropurpurea’* (**Figure 2A**), and the C:P and C:K of *P. yedoensis* Matsum were the highest (*P*< 0.05) (**Figures 2B, C**). The N:P and K:P of the petal litter of *P. yedoensis* Matsum and *P. serrulata* var. *Lannesiana* were significantly higher than those of *M. micromalus* Makino and *P. cerasifera ‘Atropurpurea’* (**Figures 2D, F**), while the N:K of *M. micromalus* Makino was substantially lower than that of *P. yedoensis* Matsum and *P. serrulata* var. *Lannesiana* (*P*< 0.05) (**Figure 2E**).

Comparative analysis of the two sample types revealed that the C:N, C:P, C:K, and K:P of the petal litters of the four Rosaceae species were higher than those of the fresh petals, as well as significant differences between the fresh petals and petal litter of *P. yedoensis* Matsum and *P. serrulata* var. *lannesiana* (*P*< 0.05) (**Figures 2A–C, F**). Meanwhile, the N:P of the petal litters of the four Rosaceae species appeared slightly lower than that of the fresh petals, with a significant difference only in *M. micromalus* Makino (*P*< 0.05) (**Figure 2D**). Besides, the N:K ratios of petal litters was significantly lower than that of the fresh petals for all four tree species (*P*< 0.05) (**Figure 2E**).

### 3.3 Nutrient resorption characteristics in the petals of four tree species

The N, P, and K resorption efficiencies of the four Rosaceae tree species were 23.87%–57.35%, 14.95%–54.55%, and 4.66%–25.40%, respectively (**Table 2**). The efficiencies in the four species were in the decreasing order of *P. yedoensis* Matsum > *P. serrulata* var. *lannesiana* > *P. cerasifera ‘Atropurpurea’* > *M. micromalus* Makino; the efficiencies of *P. yedoensis* Matsum were significantly the highest, and that of *M. micromalus* Makino was the lowest (*P<* 0.05). The N, P, and K resorption efficiencies of *P. yedoensis* Matsum were 2.40, 3.65, and 5.45 times those of *M. micromalus* Makino, respectively. Besides, the resorption efficiencies of different elements differed within the same tree species. In *P. yedoensis* Matsum and *P. cerasifera ‘Atropurpurea’*, the N and P resorption efficiencies were significantly higher than the K resorption efficiency (KRE). Besides, there were significant differences in N, P and K resorption efficiencies between *P. serrulata* var. *lannesiana* and *M. micromalus* Makino (*P*< 0.05). The order of these four tree species was NRE>PRE>KRE.

**Table 2:** N, P, and K resorption efficiencies of petals of four Rosaceae species.

Species	NRE%	PRE%	KRE%
*Prunus yedoensis* Matsum	57.35 ± 1.73Aa	54.55 ± 1.56Aa	25.40 ± 3.01Ba
*Prunus serrulata* var. *lannesiana*	33.62 ± 3.21Ab	24.49 ± 1.99Bb	11.56 ± 1.10Cb
*Malus micromalus* Makino	23.87 ± 1.16Ac	14.95 ± 2.37Bc	4.66 ± 0.25Cc
*Prunus cerasifera* ‘Atropurpurea’	24.34 ± 1.09Ac	23.69 ± 1.21Ab	7.72 ± 0.24Bbc
Average	34.80 ± 4.18	29.42 ± 4.58	12.34 ± 2.49

Uppercase letters indicate significant differences among the resorption efficiencies of different elements in the same tree species (P< 0.05), and lowercase letters indicate significant differences in the resorption efficiency of the same element among different species (P< 0.05).

### 3.4 Correlation between nutrient resorption efficiencies and nutrient contents in petals

**Figure 3:**
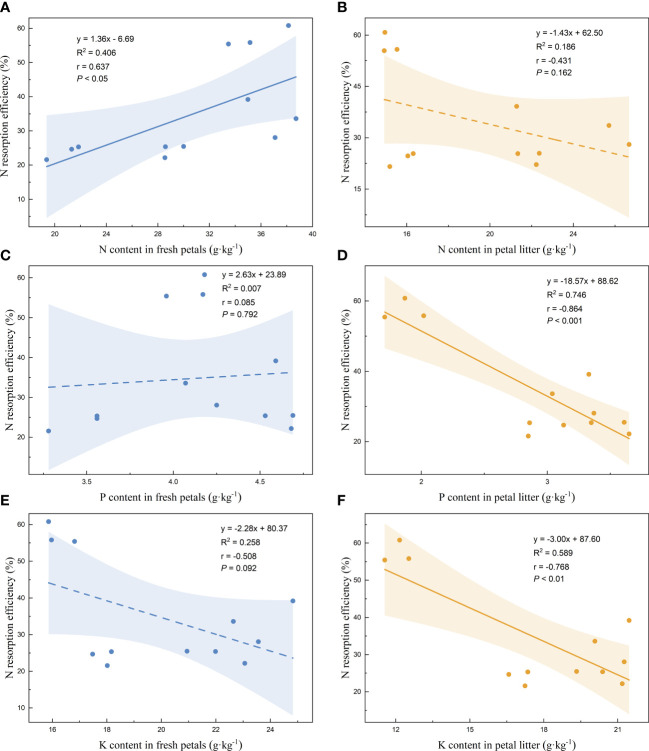
Linear fitting of N resorption efficiency of petals to the content in fresh petals and petal litter. The solid line is the significant correlation of linear regression between the two variables (*P*< 0.05), while the dashed line is the insignificant correlation (*P* > 0.05). The colored areas are 95% confidence bands.

**Figure 4:**
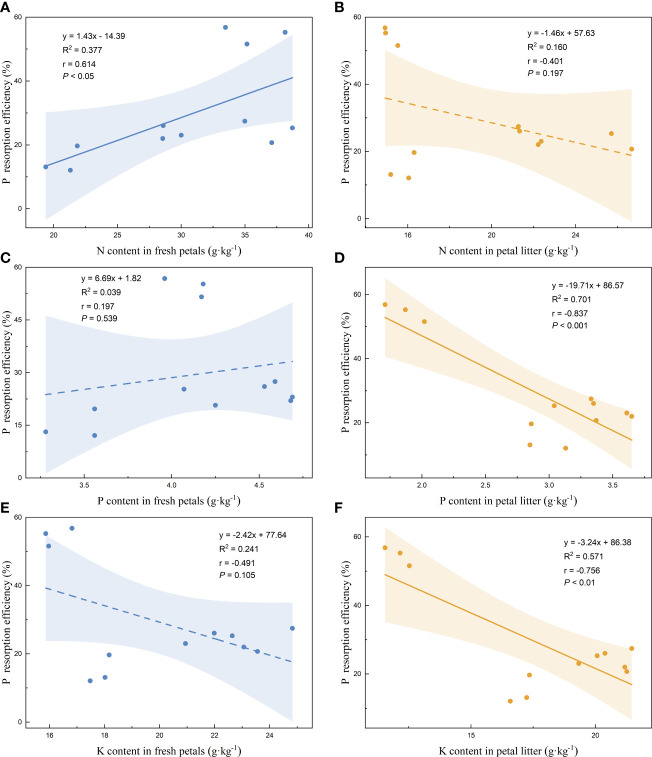
Linear fitting of P resorption efficiency of petals to the content in fresh petals and petal litter. The solid line is the significant correlation of linear regression between the two variables (*P*< 0.05), while the dashed line is the insignificant correlation (*P* > 0.05). The colored areas are 95% confidence bands.

**Figure 5:**
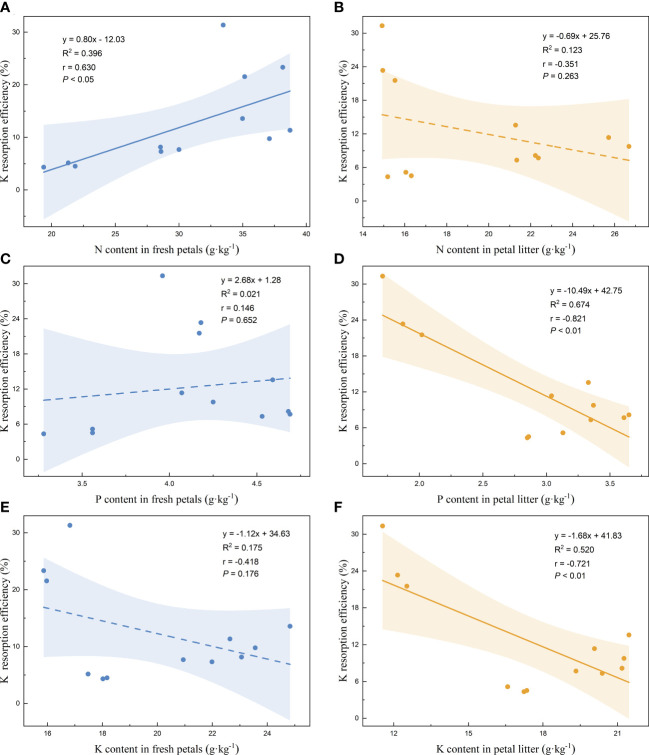
Linear fitting of K resorption efficiency of petals to the content in fresh petals and petal litter. The solid line is the significant correlation of linear regression between the two variables (*P*< 0.05), while the dashed line is the insignificant correlation (*P* > 0.05). The colored areas are 95% confidence bands.

Linear regression analysis of the nutrient resorption efficiencies of the petals and the nutrient contents of the fresh petals and petal litter was performed. The nutrient resorption efficiencies of N, P, and K of petals increased with the increase of N content in fresh petals, showing significant positive correlations (**Figures 3A**, **4A**, **5A**). NRE, PRE, and KRE were positively correlated with P content in fresh petals (**Figures 3C**, **4C**, **5C**) and negatively correlated with K content in fresh petals (**Figures 3E**, **4E**, **5E**). However, their linear regression relationships were not significant. The nutrient resorption efficiencies of N, P, and K in petals decreased with the increase of P and K contents in petal litter, showing significant negative correlations (**Figures 3D**, **4D**, **5D**, **3F**, **4F**, **5F**). However, NRE, PRE, and KRE were negatively correlated with the N content in petal litter, and the linear regression relationships between each were not significant (**Figures 3B**, **4B**, **5B**).

**Figure 6:**
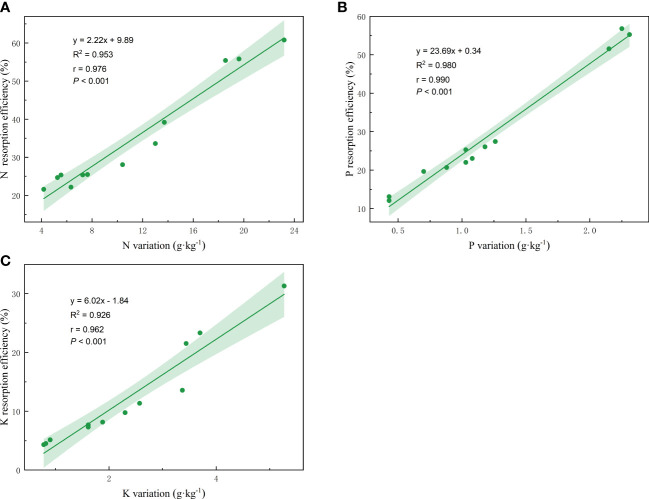
Linear fitting of N, P, K resorption efficiency and petal nutrient variation. The solid line is the significant correlation of linear regression between the two variables (*P*< 0.05). The colored areas are 95% confidence bands.

Linear regression analysis was conducted on the nutrient resorption efficiency and the nutrient variation of plants from fresh petals to petal litter, and the nutrient resorption efficiency of petal N, P, and K showed an extremely significant positive correlation with the corresponding nutrient variation (**Figure 6**). The regression equation, R^2^, r, and *P* values are as follow: y = 2.22x + 9.89, R^2 =^ 0.953, r = 0.976, *p*< 0.001 (**Figure 6A**). y = 23.69x + 0.34, R^2 =^ 0.980, r = 0.990, *p<* 0.001 (**Figure 6B**); y = 6.02x - 1.84, R^2 =^ 0.926, r = 0.962, *p<* 0.001 (**Figure 6C**).

### 3.5 Correlation between nutrient resorption efficiency and stoichiometric ratio of petals

**Figure 7:**
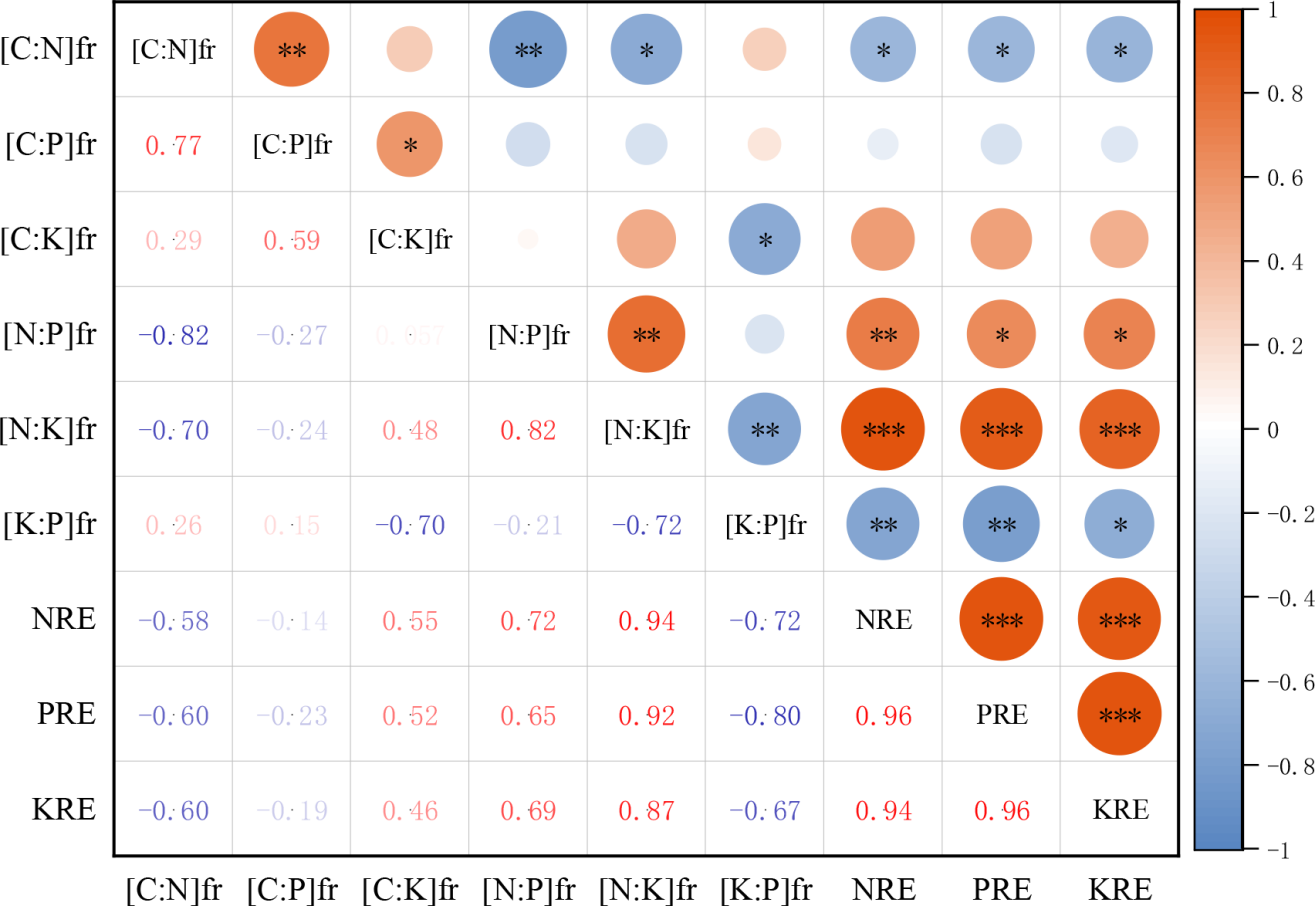
Correlation heat map of N, P, K resorption efficiency of petals with stoichiometric ratios of fresh petals. NRE is the nitrogen resorption efficiency of petal, PRE is the phosphorus resorption efficiency of petal, KRE is the potassium resorption efficiency of petal. [C:N]fr is carbon to nitrogen ratio of fresh petals, [C:P]fr is carbon to phosphorus ratio of fresh petals, [C:K]fr is carbon to potassium ratio of fresh petals, [N:P]fr is nitrogen to phosphorus ratio of fresh petals, [N:K]fr is nitrogen to potassium ratio of fresh petals, [K:P]fr is potassium to phosphorus ratio of fresh petals. *P <0.05, **P <0.01, ***P <0.001.

The correlation analysis between the stoichiometric ratio of the fresh petals and petal litter with the nutrient resorption efficiency of petals showed that the N resorption efficiency (NRE) of petals was significantly negatively correlated with the C:N of the fresh petals (*P*< 0.05). There was a significant positive correlation with the N:P and N:K of the fresh petals (*P*< 0.01, *P*< 0.001). The P resorption efficiency (PRE) of petals was positively correlated with the N:P of the fresh petals (*P*< 0.05) and negatively correlated with the K:P of the fresh petals (*P*< 0.01), but not significantly correlated with the C:P of the fresh petals (*P* > 0.05). The KRE of petals was positively correlated with the N:K of the fresh petals (*P*< 0.001), and it was negatively correlated with K:P of the fresh petals (*P*< 0.05) (**Figure 7**).

**Figure 8:**
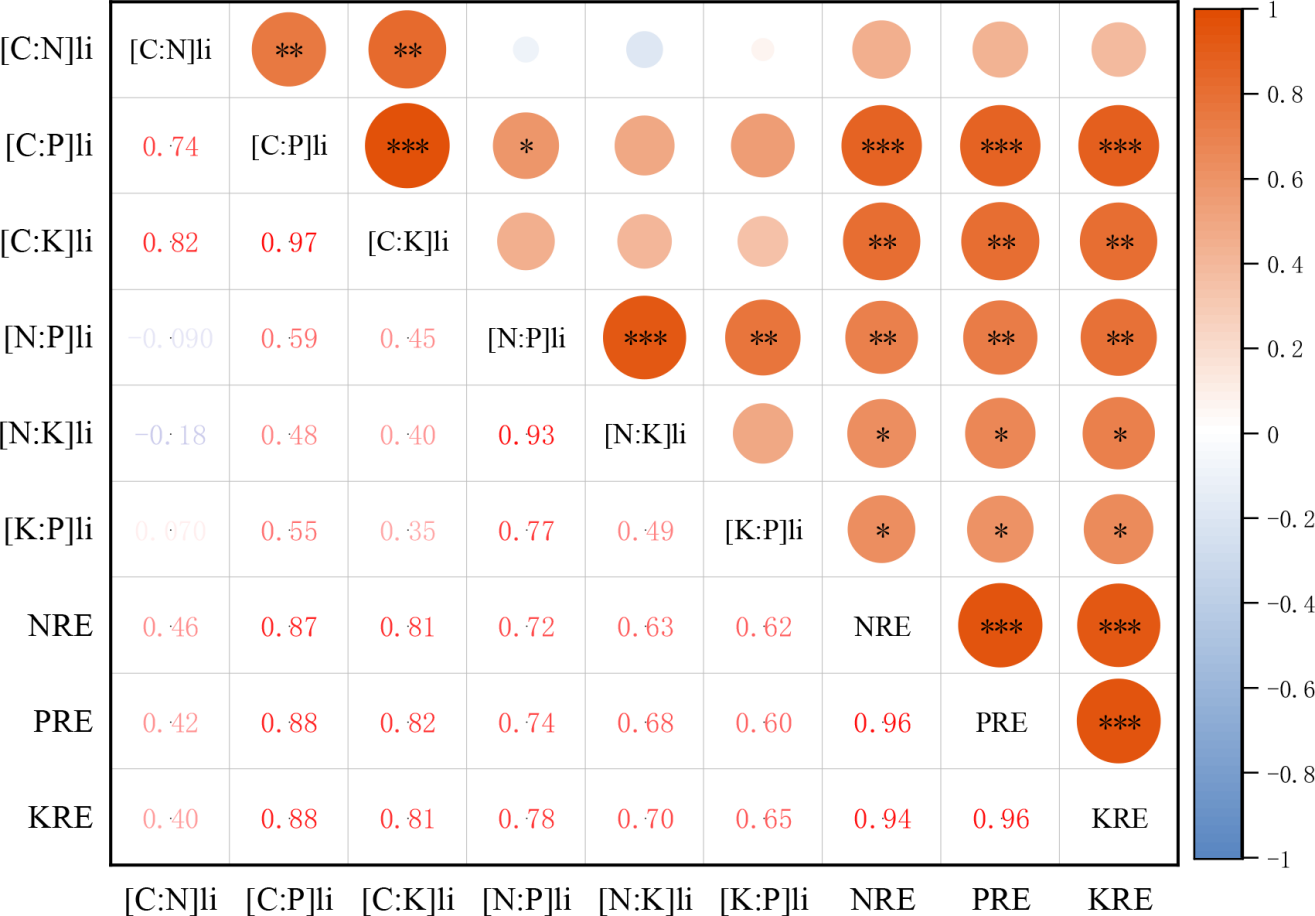
Correlation heat map of N, P, K resorption efficiency of petals with stoichiometric ratios of petal litter. NRE is the nitrogen resorption efficiency of petal, PRE is the phosphorus resorption efficiency of petal, KRE is the potassium resorption efficiency of petal. [C:N]li is carbon to nitrogen ratio of petal litter, [C:P]li is carbon to phosphorus ratio of petal litter, [C:K]li is carbon to potassium ratio of petal litter, [N:P]li is nitrogen to phosphorus ratio of petal litter, [N:K]li is nitrogen to potassium ratio of petal litter, [K:P]li is potassium to phosphorus ratio of petal litter. *P <0.05, **P <0.01, ***P <0.001.

The NRE, PRE, and KRE of petals were positively correlated with the stoichiometric ratio of petal litter. NRE of petals was significantly positively correlated with the N:P and N:K of petal litter (*P*< 0.01, *P*< 0.05), and there was no significant relationship with the C:N of petal litter (*P* > 0.05). The PRE of petals was positively correlated with the C:P, N:P, and K:P of petal litter (*P*< 0.001, *P*< 0.01, *P*< 0.05). The KRE of petals was positively correlated with the C:K, N:K, and K:P of petal litter (*P*< 0.01, *P*< 0.05, *P*< 0.05) (**Figure 8**).

## 4 Discussion

### 4.1 Nutrient content of petals of Rosaceae species

The contents of C, N, P, and K in the fresh petals of the four Rosaceae species were higher than the average C (464.00 g·kg^-1^) content of mature leaves of terrestrial plants in the world and than average contents of N (20.20 g·kg^-1^), P (1.46 g·kg^-1^) and K (15.09 g·kg^-1^) of terrestrial plants in China (Elser et al., 2000; Han et al., 2005; Qin et al., 2010) (**Figure 1**). Except for *Malus micromalus* Makino, the contents of C in the petal litter of the other three plants were lower than the average value of C (467.0 g·kg^-1^) in the litter leaves of global terrestrial plants. The N and P contents of the petal litter were higher than the global average value of N (10.0 g· kg^-1^) and P (0.7 g·kg^-1^) in the litter leaves (Yuan and Chen, 2009) and than the N (10.1 g·kg^-1^) and P (0.65 g· kg^-1^) contents of the litter leaves in eastern China (Tang et al., 2013). The K content of petal litter was also higher than that of litter leaves of woody plants in northern China (Zhang, 2018) (**Figure 1**). The physiological functions of plants, as well as the distribution of nutrient elements of different organs, are different. Usually, large amounts of nutrient elements are invested in the parts with the strongest physiological functions (Liu et al., 2008; Jiang, 2016). It has been found that the C content of plant reproductive organs is higher than other organs (Zheng et al., 2007). In a study of *Ceratocephalus orthoceras*, it was found that a large amount of P was transferred from other organs to floral organs before flowering (Jia et al., 2013). Ma et al. (2009) found K distribution of wild *Cerasus humilis* floral organs to be greater than that of other tissues and organs. In the present study, the contents of C, N, P, and K of the fresh petals were higher than those in mature leaves of terrestrial plants in the world and China. This may be due to the selected tree species all growing leaves after flowering plants and flowers were the reproductive organs of plants. The good development of flowers and fruits is the material basis for the growth of leaves and other vegetative organs, and plants may have the principle of priority of reproductive organs in nutrient supply strategies. In order to meet the needs of reproduction, plants tend to prioritize nutrient needs of reproductive organs such as flowers in nutrient absorption and distribution strategies. The contents of N, P, and K in the four Rosaceae species were higher in fresh petals than in petal litter, indicating that the petals transferred some nutrients to other tissues before falling, resulting in the nutrient content in the petal litter was lower than that in the fresh petals, which verified that the petals had a similar leaf resorption process. Nutrient resorption mechanism is a survival strategy for plants to cope with nutrient poor soil. There was a nutrient resorption process in the petals of small trees of Rosaceae, but whether the petals of herbaceous plants, vines and shrubs have the same process require further studies.

### 4.2 Stoichiometric characteristics of petals of Rosaceae species

The stoichiometric ratio of plants can reflect their nutrient utilization efficiency, C assimilation efficiency, and nutrient limitation, which has important ecological significance (Aerts and Chapin, 1999; Güsewell, 2004; Yan et al., 2010). The C:N, C:P, and N:P of the fresh petals of the four Rosaceae species were significantly lower than the average values of C:N (30.9), C:P (374.7) and N:P (12.6) of mature leaves of terrestrial plants at global scale (Elser et al., 2000; Zeng et al., 2013), and the N:P ratio of the fresh petals was also lower than that of terrestrial plants in China (14.4) (Ren et al., 2007; Zeng et al., 2013) (**Figure 2**). The C:N, C:P and N:P of petal litter of the four Rosaceae species were significantly lower than the mean values of C:N (50.1), C:P (659.0) and N:P (13.1) of leaf litter in the global temperate broad-leaved forest (McGroddy et al., 2004). It was also significantly lower than the average C:N (44.8), C:P (1132.5) and N:P (25.0) of leaf litter in Chinese forest ecosystems (Wang et al., 2011) (**Figure 2**). Some researchers have proposed that the C:N and C:P of fresh leaves could be used to characterize the ability of plants to absorb N and P nutrient elements while assimilating C, reflecting the C fixation efficiency and N and P utilization efficiency of plants (He and Dijkstra, 2014). The C:N and C:P of the four Rosaceae species were lower than those of the global and Chinese horizontal leaves, indicating that the nutrient utilization efficiency of plant petals was lower than that of leaves. Among the four Rosaceae plants, the C:N and C:P of *Malus micromalus* Makino of the fresh petals were significantly higher, indicating that it had higher N, P utilization efficiency and carbon assimilation efficiency than the other three species. Generally, the C:N of leaf litter was negatively correlated with the decomposition rate of litters and that lower C:N ratio and higher initial N concentration were usually associated with a faster decomposition rate. Some scholars have also found that the initial N content in leaf litter can indicate the mass loss of litter. The decomposition rate of leaf litter is negatively correlated with the initial C content and has no significant correlation with litter C:N but has significant positive correlation with the initial N concentration (Chen et al., 2005). Ma et al. (2021) found the decomposition rate of petal litter to be positively correlated with the content of N, P, and K, and negatively correlated with N:P in the decomposition efficiency of fallen flowers of common trees in northern cities of China. There are some differences in the research results, which may be related to different species and different litter properties. Compared with the other three plants, the petal litter of *Prunus cerasifera* ‘Atropurpurea’ contain higher N, P, K content and lower N:P, so the petal litter of *Prunus cerasifera* ‘Atropurpurea’ are relatively easier to decompose. The N:P of fresh leaf has often been used to determine the element limitation during plant growth and reproduction (Dijkstra et al., 2012). It is generally believed that when the N:P of fresh leaf<14, plant growth is mainly restricted by N. When the N:P of fresh leaves >16, plant growth was mainly limited by P. When fresh leaves 14<N:P<16, plants are restricted by both N and P (Koerselman and Meuleman, 1996). In addition, Güsewell (2004) proposed that when the N:P<10 or N:P >20, the leaves can be determined N limitation or P limitation. Some researchers also proposed that when the leaves were N:P<14.5 and N:K<2.1, plant growth was limited by N; when N:P>14.5 and K:P>3.4, plant growth was limited by P or P and K; when N:K>2.1 and K:P<3.4, growth was limited by K or K and N (Venterink et al., 2003; Wang et al., 2019). However, there is a lack of research on the threshold of nutrient restriction in flower petals, which needs further exploration in the future.

### 4.3 Nutrient resorption characteristics of petals of Rosaceae species

Vergutz et al. (2012) studied terrestrial plant leaves at the global scale and found that approximately 62.1% N, 64.9% P, and 70.0% K were reabsorbed and transferred to other tissues (Lu and Yang, 2020). The NRE, PRE, and KRE of the petals of the four Rosaceae species were significantly lower than the nutrient resorption efficiency of the leaf at the global level (**Table 2**). It indicates that the proportion of nutrients transferred from petals to other tissues of plants during falling is smaller than that of leaves. The contents of N, P, and K in the petal litter of *Prunus yedoensis* Matsum were significantly lower than those of the other three tree species, so the nutrient resorption potential of *Prunus yedoensis* Matsum was higher. At the same time, the study on the resorption efficiency of N, P, and K in the petals of the four plants also found that *Prunus yedoensis* Matsum > *Prunus serrulata* var. *lannesiana* > *Prunus cerasifera* ‘Atropurpurea’ > *Malus micromalus* Makino, indicating that *Prunus yedoensis* Matsum can better adapt to the harsh and barren environment. Tang (2012) and Han et al. (2013) believed that plants tend to absorb more nutrient that limits their growth. In other word, plants tend to absorb more restricted elements that is “relative nutrient resorption”. When RR≈0, plants are limited by N, P balance or common restrictions; when RR>0, the plant NRE was greater than PRE, and the plant recovered a larger proportion of N, so the plant growth was limited by N; when RR<0, plants recovered a greater proportion of P, and plant growth was limited by P. In this study, the resorption efficiency of N, P, and K elements of petals in four Rosaceae species was NRE>PRE>KRE (RR>0), indicating that compared with P and K elements, petals recovered a larger proportion of N element before falling, and the growth of plants during the flowering process was mainly limited by N element. Therefore, more nitrogen fertilizer should be applied to the maintenance and management of the four Rosaceae plants throughout the flowering period to ensure the growth and development of the plants. However, efficient NRE may be a strategy for the four Rosaceae plants to adapt to a low N environment.

### 4.4 Effects of petal nutrient content and stoichiometry on nutrient resorption efficiency

Nutrient resorption efficiency is affected by multiple factors, including leaf and soil nutrients, growth environment and climatic conditions (Zhou et al., 2020). We found that the resorption efficiencies of N, P, and K in Rosaceae were positively correlated with the content of N in fresh petals. It was contrary to the finding of Kobe et al. (2005). They found that the nutrient resorption efficiency of N and P in leaves was significantly negatively correlated with the content of N and P in mature leaves. However, some other studies have shown that nutrient resorption efficiency had no or very weak relationship with the corresponding element content in mature leaves (Chapin and Kedrowski, 1983; Escudero et al., 1992; Aerts, 1996). According to the mechanism of plant nutrient resorption, the smaller the nutrient content of the plant itself, the more nutrients need to be reabsorbed to redress the deficiency of its own nutrients. Therefore, the negative correlation between nutrient resorption efficiency and nutrient content of fresh petals is the most consistent result of this mechanism.

We found that the resorption efficiencies of N, P, and K in petals were negatively correlated with the contents of P and K in litter of petals. Killingbeck (1996) proposed using nutrient concentration of leaf litter to characterize the nutrient resorption capacity of plants. The lower the nutrient concentration of leaf litter, the higher the proportion of plant nutrient resorption. There was a significant positive correlation between the nutrient resorption efficiency of petals and the amount of nutrient variation, which indicated that the nutrient resorption of petals before falling down was one of the important factors determining the nutrient resorption efficiency.

The NRE of petals was negatively correlated with the C:N of fresh petals and positively correlated with the N:P and N:K of fresh petals. The PRE was positively correlated with N:P and negatively correlated with K:P of fresh petals. The KRE of petals was positively correlated with N:K and negatively correlated with K:P of fresh petals. The nutrient resorption efficiency of petal N, P, and K was positively correlated with the stoichiometric ratios of petal litter. Overall, the correlation between the nutrient resorption efficiency of petals and the stoichiometric ratio of petal litter was more obvious. Similar to results of Wang et al. (2019) on the relationship between nutrient resorption and stoichiometric ratio in alfalfa leaves, the nutrient resorption efficiency of leaves was basically positively correlated with the stoichiometric ratio of senescent leaves. However, unlike in the study of nutrient resorption and C:N:P stoichiometric characteristics of *Eucalyptus grandis × urophylla* at different ages by Qiu et al. (2017), they found that NRE was significantly positively correlated with C:N of senescent leaves and that PRE was significantly negatively correlated with C:P of fresh leaves. This also differs from the results in Lu and Yang (2020) on forage grass in the Loess Plateau, which found that PRE had a good correlation with N, P, and K stoichiometric characteristics of leaves, while NRE and KRE had a poor correlation with stoichiometric ratios. N:P and N:K of petals were closely correlated with nutrient resorption efficiency, while C:N, C:P and C:K were negatively correlated or not correlated with carbon participation, indicating poor carbon reuse and that nitrogen was significantly recovered. This may be related to structural or organic materials produced by carbon and nitrogen in the petals.

The different correlation conclusions between plant nutrient resorption and stoichiometric ratio are mainly due to differences in species, experimental area conditions, and measurement methods (Kobe et al., 2005). At present, no clear conclusion has been drawn on the internal relationship between plant nutrient resorption and C:N:P stoichiometry, and there is a need for more quantitative research on the internal relationship between the two (Kobe et al., 2005; Qin et al., 2017).

## 5 Conclusions

There were interspecific differences in C, N, P, and K elements content, stoichiometric ratio and nutrient resorption efficiency of the fresh petals and petal litter of the four Rosaceae species. The nutrient contents of petal litter were lower than those of the fresh petals; thus, petals had transferred nutrients to other tissues of the plant before fall, and the nutrient resorption process of petals was similar to that of leaves. The nutrient content of petals was higher than that of leaves at global level, but the stoichiometric ratio and nutrient resorption efficiency of petals were lower than those of leaves at the global level. The N and P utilization efficiencies and carbon assimilation efficiency of *Malus micromalus* Makino were higher. The petal litter of *Prunus cerasifera* ‘Atropurpurea’ contains higher contents of N, P, and K and lower N:P, so its petal litter was relatively easier to decompose. *Prunus yedoensis* Matsum has higher N, P, and K resorption efficiencies and better adaptability to the environment. NRE was all higher than PRE and KRE in each of the four plants, and petals recovered a larger proportion of N element before falling. The growth of plants during flowering was mainly restricted by N element. Therefore, N fertilizer could be applied before flowering to ensure good growth and development. The resorption efficiency of N, P, and K in petals was positively correlated with the content of N in fresh petals, and negatively correlated with the content of P and K in petal litter. In addition, the nutrient resorption efficiency of petals was positively correlated with nutrient variation. The correlation between the nutrient resorption efficiency of petals with nutrient content and stoichiometric ratio of petal litter was stronger.

This work is the first on plant nutrient resorption and stoichiometric ratio in reproductive organs such as petals, which defines the nutrient requirements and nutrient limitations of the growth and development of flowering trees in urban green space, as well as providing a scientific basis and theoretical support for the utilization and nutrient management of urban landscaping trees. However, only the effects of plant nutrient status on plant nutrient resorption, and soil nutrient were considered; moisture content, plant functional traits, temperature, light, and other environmental factors are also important factors affecting plant nutrient resorption, requiring further studies. The regulation mechanism of petal nutrient resorption characteristics and stoichiometric characteristics, such as the threshold range of limiting elements, remain unclear, and a large number of experiments are needed for further verification.

## Data availability statement

The original contributions presented in the study are included in the article/supplementary material. Further inquiries can be directed to the corresponding author.

## Author contributions

YL and DS designed experiments and initiated research projects. YL, DS, SL, LF, JY, and YX performed experiments and analyzed data. All authors contributed to the writing of the manuscript. All authors contributed to the article and approved the submitted version.
